# Methodological Quality of Systematic Reviews on Platelet-Rich Plasma Therapy for Osteoarthritis: A Meta-Research Study

**DOI:** 10.1055/s-0045-1802964

**Published:** 2025-04-28

**Authors:** Maria Eduarda Oliveira Onuki, Kamilla Mayr Martins Sá, Marcela Lourenço Alves, Maria Eduarda de Souza, Elaine Marcílio Santos, Ana Luiza Cabrera Martimbianco

**Affiliations:** 1 Faculdade de Medicina, Universidade Metropolitana de Santos, Santos, SP, Brasil; 2 Programa de Pós-graduação em Saúde e Meio Ambiente, Universidade Metropolitana de Santos, Santos, SP, Brasil; 3 Centro de Avaliação de Tecnologias em Saúde, Hospital Sírio-Libanês, São Paulo, SP, Brasil

**Keywords:** osteoarthritis, platelet-rich plasma, systematic review

## Abstract

**Objective** To assess the methodological quality of systematic reviews on the benefits and disadvantages of platelet-rich plasma (PRP) to treat osteoarthritis.

**Methods** We conducted a comprehensive literature search, and the methodological quality of the included reviews was assessed using the tool A Measurement Tool to Assess Systematic Reviews, Version 2 (AMSTAR-2). In addition, the assessment of the certainty of evidence using the Grading of Recommendations Assessment, Development, and Evaluation (GRADE) approach was investigated. A total of 31 systematic reviews met the inclusion criteria.

**Results** Almost 84% of the articles received an overall rating of critically-low quality, and 16.1%, of low quality. The methodological criteria most frequently reported in an inadequate manner were related to search strategies, with 77.3% of “no” or “partially yes” responses, and the reasons to exclude studies, which were not described in 100% of the reviews. Furthermore, 42% did not mention the registration of their protocols, 13% did not use the appropriate methodological quality tool to assess the risk of bias of the included clinical trials, 45.2% did not consider the risk of bias in the discussion of the results, and 32.26% did not report or planned to report publication bias. The GRADE approach was only used in 19.3% of the reviews.

**Conclusion** Despite the high number of systematic reviews on PRP for osteoarthritis, in the present meta-research study, we identified that most are not conducted adequately, presenting methodological flaws that may affect the reliability of clinical findings.

## Introduction

Health interventions often gain popularity and become the focus of widespread scientific investigation. This is often the ideal scenario for the proliferation of evidence syntheses derived from the results of the numerous clinical trials published on the efficacy and safety of these interventions. A notable example is platelet-rich plasma (PRP) therapy, 1 which involves injecting a solution with a high concentration of an individual's platelets, which is considered promising due to its potential to release growth factors, cytokines, and various substances that induce analgesia, anti-inflammatory, and tissue anabolic effects at the site of application, resulting in a possible renewal of damaged tissue. 2 3 4 In addition, it is a minimally-invasive and easily-accessible technique which has received significant attention in recent years due to the potential benefit of adjuvant treatment for various clinical conditions. In particular, the growing interest in the use of PRP in the treatment of osteoarthritis is highlighted by the importance of synovial inflammation in the pathophysiology and its high prevalence worldwide. 4 5

Given the significant increase in scientific publications on this topic, as expected, the number of systematic reviews on the effects of PRP for osteoarthritis has increased progressively in the last ten years, resulting in a mass of evidence synthesis that often disregards methodological standards and recommendations, compromising the results. 6 7 Given this scenario, meta-research studies emerge as an important tool to establish a connection between good science and its applicability in the clinical practice. 8 9 By gathering and evaluating methodological appropriateness in planning, conducting, and reporting different study designs, meta-research provide information about how common and harmful are certain biases to a research field. 10 In addition to identifying methodological flaws produced and reproduced in the numerous systematic reviews on PRP for osteoarthritis, a meta-research study can direct future efforts toward developing more pertinent and high-quality reviews in this field, increasing the transparency and reproducibility of their findings. 11 12

Thus, the current meta-research study aimed to evaluate the methodological quality of systematic reviews on the benefits and disadvantages of PRP compared to placebo, to other interventions, or to no intervention in the treatment of patients with osteoarthritis.

## Materials and Methods

The present study followed the methodological guidance recommended for meta-research studies. 13 To improve reporting quality, the relevant items from the Preferred Reporting Items for Systematic Reviews and Meta-Analyses (PRISMA) statement 14 were used. The study protocol was registered on the Open Science Framework (available at: https://doi.org/10.17605/OSF.IO/NZXT9 ).

### Criteria to Include Systematic Reviews

Studies identified as systematic reviews on the effects of PRP in the treatment of any type of osteoarthritis were eligible for inclusion. Only those explicitly labelled as “systematic reviews” were considered, while review protocols and systematic reviews incorporating observational study designs were excluded. Additionally, systematic reviews available only as abstracts or in incomplete formats were excluded.

### Information Sources

To identify eligible systematic reviews, we conducted a comprehensive search on January 31, 2024, on the following electronic databases: Medical Literature Analysis and Retrieval System Online (MEDLINE, via Pubmed), The Cochrane Library (Cochrane Database of Systematic Reviews, CDSR), Excerpta Medica Database (Embase, via Elsevier), and Epistemonikos. No restrictions on publication date or language were applied. The detailed search strategies are presented in Table 1 .

**Table 1:** Search strategies

Database	Search strategies	Results
Medical Literature Analysis and Retrieval System Online (MEDLINE, via Pubmed)	#1 “Osteoarthritis” [Mesh] OR Osteoarthritides OR Osteoarthrosis OR Osteoarthroses OR (Arthritis, Degenerative) OR (Arthritides, Degenerative) OR (Degenerative Arthritides) OR (Degenerative Arthritis) OR Arthrosis OR Arthroses #2 “Platelet-Rich Plasma” [Mesh] OR (Plasma, Platelet-Rich) OR (Platelet Rich Plasma) OR PRP #3 systematic[sb] #4 #1 AND #2 AND #3	221
The Cochrane Library (Cochrane Database of Systematic Reviews, CDSR)	#1 MeSH descriptor: [Osteoarthritis] explode all trees #2 Osteoarthritides OR Osteoarthrosis OR Osteoarthroses OR (Arthritis, Degenerative) OR (Arthritides, Degenerative) OR (Degenerative Arthritides) OR (Degenerative Arthritis) OR Arthrosis OR Arthroses #3 MeSH descriptor: [Platelet-Rich Plasma] explode all trees #4 (Plasma, Platelet-Rich) OR (Platelet Rich Plasma) OR PRP #5 #1 OR #2 #6 #3 OR #4 #7 #5 AND #6 In Cochrane Reviews	7
Excerpta Medica Database (Embase, via Elsevier)	#1 'osteoarthritis'/exp OR Osteoarthritides OR Osteoarthrosis OR Osteoarthroses OR 'Arthritis, Degenerative' OR 'Arthritides, Degenerative' OR 'Degenerative Arthritides' OR 'Degenerative Arthritis' OR Arthrosis OR Arthroses #2 'platelet-rich plasma cell'/exp OR 'Plasma, Platelet-Rich' OR 'Platelet Rich Plasma' OR PRP #3 ('systematic review' OR 'meta-analysis') AND [review]/lim OR 'meta analysis'/exp OR 'meta analysis' OR 'systematic review'/exp OR 'systematic review' OR 'systematic review (topic)'/exp OR 'systematic review (topic)' OR 'meta analysis (topic)'/exp OR 'meta analysis (topic)' OR 'biomedical technology assessment'/exp OR 'biomedical technology assessment' OR 'network meta-analysis'/exp OR 'network meta-analysis' OR ((systematic* NEAR/3 (review* OR overview*)):ti,ab,kw) OR ((methodologic* NEAR/3 (review* OR overview*)):ti,ab,kw) OR ((quantitative NEAR/3 (review* OR overview* OR synthes*)):ti,ab,kw) OR ((research NEAR/3 (integrati* OR overview*)):ti,ab,kw) OR ((integrative NEAR/3 (review* OR overview*)):ti,ab,kw) OR ((collaborative NEAR/3 (review* OR overview*)):ti,ab,kw) OR ((pool* NEAR/3 analy*):ti,ab,kw) OR 'data synthes*':ti,ab,kw OR 'data extraction*':ti,ab,kw OR 'data abstraction*':ti,ab,kw OR 'handsearch*':ti,ab,kw OR 'hand search*':ti,ab,kw OR 'mantel haenszel':ti,ab,kw OR 'peto':ti,ab,kw OR 'der simonian':ti,ab,kw OR 'dersimonian':ti,ab,kw OR 'fixed effect*':ti,ab,kw OR 'latin square*':ti,ab,kw OR 'met analy*':ti,ab,kw OR 'metanaly*':ti,ab,kw OR 'technology assessment*':ti,ab,kw OR 'hta':ti,ab,kw OR 'htas':ti,ab,kw OR 'technology overview*':ti,ab,kw OR 'technology appraisal*':ti,ab,kw OR 'meta regression*':ti,ab,kw OR 'metaregression*':ti,ab,kw OR 'meta-analy*':ti,ab,kw,ok OR 'metaanaly*':ti,ab,kw,ok OR 'systematic review*':ti,ab,kw,ok OR 'biomedical technology assessment*':ti,ab,kw,ok OR 'bio-medical technology assessment*':ti,ab,kw,ok OR medline:ti,ab,ok OR cochrane:ti,ab,ok OR pubmed:ti,ab,ok OR medlars:ti,ab,ok OR embase:ti,ab,ok OR cinahl:ti,ab,ok OR cochrane OR 'health near/2 technology assessment' OR 'evidence report' OR ((comparative NEAR/3 (efficacy OR effectiveness)):ti,ab,kw,ok) OR 'outcomes research':ti,ab,kw,ok OR 'relative effectiveness':ti,ab,kw,ok OR ((('indirect' OR 'indirect treatment' OR 'mixed-treatment' OR 'bayesian') NEAR/3 comparison*):ti,ab,kw,ok) OR 'meta-analysis'/dm OR 'systematic review'/dm OR ((multi* NEAR/3 treatment NEAR/3 comparison*):ti,ab,kw,ok) OR ((mixed NEAR/3 treatment NEAR/3 ('meta analy*' OR metaanaly*)):ti,ab,kw,ok) OR (umbrella:ti,ab,kw,ok AND review*:ti,ab,kw,ok) OR ((multi* NEAR/2 paramet* NEAR/2 evidence NEAR/2 synthesis):ti,ab,kw,ok) OR ((multiparamet* NEAR/2 evidence NEAR/2 synthesis):ti,ab,kw,ok) OR (('multi paramet*' NEAR/2 evidence NEAR/2 synthesis):ti,ab,kw,ok) #4 #1 AND #2 AND #3 AND [embase]/lim NOT ([embase]/lim AND [medline]/lim)	135
Epistemonikos	((title:(Osteoarthritis OR Osteoarthritides OR Osteoarthrosis OR Osteoarthroses OR (Arthritis, Degenerative) OR (Arthritides, Degenerative) OR (Degenerative Arthritides) OR (Degenerative Arthritis) OR Arthrosis OR Arthroses) OR abstract:(Osteoarthritis OR Osteoarthritides OR Osteoarthrosis OR Osteoarthroses OR (Arthritis, Degenerative) OR (Arthritides, Degenerative) OR (Degenerative Arthritides) OR (Degenerative Arthritis) OR Arthrosis OR Arthroses))) OR abstract:((title:(Osteoarthritis OR Osteoarthritides OR Osteoarthrosis OR Osteoarthroses OR (Arthritis, Degenerative) OR (Arthritides, Degenerative) OR (Degenerative Arthritides) OR (Degenerative Arthritis) OR Arthrosis OR Arthroses) OR abstract:(Osteoarthritis OR Osteoarthritides OR Osteoarthrosis OR Osteoarthroses OR (Arthritis, Degenerative) OR (Arthritides, Degenerative) OR (Degenerative Arthritides) OR (Degenerative Arthritis) OR Arthrosis OR Arthroses)))) AND (title:((Plasma, Platelet-Rich) OR (Platelet Rich Plasma) OR PRP) OR abstract:((Plasma, Platelet-Rich) OR (Platelet Rich Plasma) OR PRP))	122

### Study Selection

A pair of reviewers (MEO and MLA) analyzed the title and abstract of the references obtained with the search strategy. This initial screening was performed through the Rayyan (Qatar Computing Research Institute, Doha, Qatar) application, 15 which enabled the evaluation process to occur independently. After that first stage, the full text of the studies classified as potentially included was thoroughly examined. Any conflicts in both stages were resolved through a third reviewer. The excluded studies were recorded in the table of excluded studies, accompanied by the respective reasons for exclusion ( Table 2 ). Two independent reviewers used a pre-established data extraction form to collect data from the included studies. Following this process, a third reviewer (ALCM) resolved potential inconsistencies.

**Table 2:** List of excluded studies and reasons for exclusion

Systematic Review	Justification
**Belk, 2023**	Associated PRP with another intervention (BMAC)
**Chou, 2021**	Included retrospective studies
**Derwich, 2021**	Associated PRP with another intervention
**Evans, 2020**	Included case series
**Hegazy, 2019**	Included cohort studies
**Howlader, 2023**	Included cohort studies
**Kim, 2021**	Included cohort studies
**McLarnon, 2021**	Included cohort studies
**Paget, 2023**	Trials without PRP
**Patel, 2022**	Included prospectives and retrospectives studies
**Tietze, 2015**	Included case series

Abbreviations: BMAC, bone marrow aspirate concentrate; PRP, platelet-rich plasma.

### Data Extraction

For eligible systematic reviews, we extracted the following information: year of publication, number of included studies, aspects of the intervention and comparators, review protocol, tool used to assess methodological quality, outcomes, if meta-analyses were conducted, and funding sources. The authors evaluated several systematic reviews that assessed the certainty of the body of the evidence using the Grading of Recommendations Assessment, Development, and Evaluation (GRADE) approach 16 and the adequacy of the protocol registration regarding selective reporting bias.

### Methodological Quality Evaluation of the Included Studies

We used A Measurement Tool to Assess Systematic Reviews, Version 2 (AMSTAR-2) tool 17 to critically assess the methodological quality of the systematic reviews. The tool has 16 items, and after an overall assessment of the systematic reviews, the study was categorized into four quality levels: critically-low, low, moderate, and high, depending on the weaknesses detected in critical and non-critical items. Two independent reviewers (KMMS and MES) conducted the AMSTAR-2 assessment; a third reviewer (EMS) was consulted in case of disagreements.

### Synthesis of the Results

The methodological characteristics of the included systematic reviews were tabulated and summarized descriptively using a Microsoft Excel (Microsoft Corp., Redmond, WA, United States) spreadsheet. Data were expressed as absolute and relative frequencies.

## Results

### Search Results

**Fig. 1:**
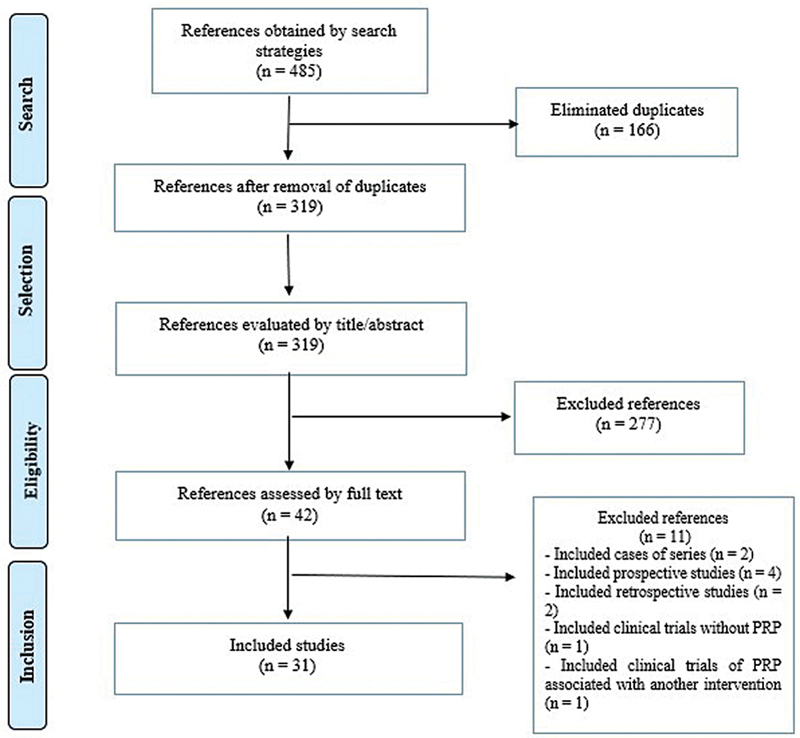
Flowchart of the study selection process.

With the search strategies, we initially retrieved 485 references. After eliminating 166 duplicates, 319 references underwent title and abstract screening. Thus, 31 systematic reviews met the eligibility criteria and were included in the present meta-research study ( Fig. 1 ).

### Baseline Characteristics

Table 3 presents the detailed characteristics of the included systematic reviews, which assessed between 3 and 40 randomized clinical trials (RCTs) on the effects of PRP compared to placebo or other therapies. These reviews were published from 2015 to 2023, with the highest number of study publications in 2020. 18 19 20 21 22 23 24

**Table 3:** Main characteristics of the included systematic reviews

First author, year	Included RCTs (n)	Interventions versus (vs.) comparators	Systematic review protocol	Methodological quality assessment	Outcomes	Meta-analysis	Funding sources
Ali, 2018	3	PRP vs. HA; PRP vs. PRP + HA	Not registered	Jadad 18	Pain and function	No	No funding
Belk, 2020	18	PRP vs. HA	Not registered	Cochrane RoB tool 19 and Modified Coleman Methodology Score 20	Pain, function, and LP-PRP vs. LR-PRP adverse events	Yes	No funding
Cavazos, 2019	5	Single injection of PRP vs. multiple injections of PRP	Registered on PROSPERO (CRD42018106429)	Cochrane RoB tool 19	Pain and Function	Yes	No funding
Chen, 2020	14	PRP vs. HA	Not registered	Cochrane RoB tool 19	Pain, function, and adverse events	Yes	No funding
Chung, 2018	5	PRP vs. placebo; PRP vs. HA; PRP vs. saline solution	PROSPERO register (number not reported)	Cochrane RoB tool 19	Pain and function	Yes	No funding
Costa, 2022	40	PRP vs. HA; PRP vs. corticosteroid; PRP vs. saline solution; PRP vs. exercise; PRP vs. oral pharmacological agents; PRP vs. ozone therapy; PRP vs. placebo	Registered on PROSPERO (CRD42018093247)	Cochrane RoB tool 19	Pain, function, failure of treatment, and adverse events	Yes	No funding
Dai, 2017	10	PRP vs. HA; PRP vs. saline solution; PRP vs. corticosteroid; PRP vs. exercise; PRP vs. no treatment	Not registered	Cochrane RoB tool 19	Pain, function, and adverse effects	Yes	No funding
Di, 2018	7	PRP vs. HA	Registered on PROSPERO (CRD42016048394)	Cochrane RoB tool 19	Pain and function	No	No funding
Filardo, 2020	34	PRP vs. placebo; PRP vs. HA; PRP vs. saline solution; PRP vs. CS; PRP vs. ozone	Registered on PROSPERO (CRD42019145409)	Cochrane RoB tool 2.0 21	Pain, function, and stiffness	Yes	No funding
Garcia, 2020	7	PRP vs. HA; PRP vs. PRP + HA; PRP vs. saline solution; PRP vs. bupivacaine; PRP vs. no injection	Registered on PROSPERO (CRD42020159802)	Coleman Methodology Score 20	Pain, function, and return to daily activities and sports	Yes	No funding
Gong, 2021	6	PRP vs. HA	Registered on PROSPERO (CRD42020182571)	Cochrane RoB tool 19	Pain, function, and adverse events	Yes	National Natural Science Foundation of China; Scientific Research Project of Chinese Academy of Traditional Chinese Medicine
Hohmann, 2020	12	PRP vs. HA	Not registered	Cochrane RoB tool 1 19	Pain and function	Yes	No funding
Hong, 2021	23	PRP vs. placebo; PRP vs. HA; PRP vs. CS; PRP vs. oral NSAIDs	Not registered	Modified version of the Jadad Scale 22	Pain, function, and adverse events	Yes	Beijing Municipal Science and Technology Commission, the National Natural Science Foundation of China, the National Key Research and Development Program of China, the Capital Health Research and Development of Special
Idres, 2023	9	PRP vs. corticosteroids	Not registered	Cochrane RoB tool 19	Pain, function, and stiffness	No	No funding
Kanchanatawan, 2015	9	PRP vs. HA; PRP vs placebo	Not registered	PRISMA 14	Pain, function, stiffness, and adverse events	Yes	No funding
Kim, 2023	21	PRP vs. HA	PROSPERO (register number not reported)	Cochrane RoB tool 19 and Modified Coleman Methodology Score 20	Pain, function, stiffness, and adverse events	Yes	No funding
Meheux, 2016	6	PRP vs. corticosteroids; PRP vs. HA; PRP vs. placebo	Registered on PROSPERO (CRD42014013032)	Modified Coleman Methodology Score 23	Pain, function, and stiffness	No	No funding
Nie, 2021	21	PRP vs. corticosteroids; PRP vs. HA; PRP vs. placebo	Registered on PROSPERO (CRD42019122002)	Cochrane RoB tool 19	Pain, adverse events, and complications	Yes	No funding
Peng, 2021	14	PRP vs. HA	Registered on PROSPERO (CRD42022347244)	Cochrane RoB tool 19	Pain, function, and adverse events	Yes	No funding
Porqueres, 2020	4	PRP vs. HA	Registered on PROSPERO (CRD42014010210)	Cochrane RoB tool 19	Pain, function, growth factors, and adverse events	Yes	No funding
Sadabad, 2016	6	PRP vs. HA	Not registered	Cochrane RoB tool 19 and Jadad 18	Pain	Yes	Health Technology Assessment Department at Shahid Sadoughi University of Medical Sciences, Yazd
Sambe, 2023	7	PRP vs. HA	Not registered	Cochrane RoB tool 2.0 21	Pain, function, and adverse events	Yes	No funding
Sax, 2022	24	PRP vs. CS; PRP vs. HA; PRP vs. saline solution and exercise therapy	Registered on PROSPERO (CRD42022296909)	Modified Coleman Methodology Score 23	Pain	Yes	No funding
Shang, 2023	14	PRP vs. HA	Not registered	Jadad 18	Pain	Yes	No funding
Shen, 2017	14	PRP vs. HA; PRP vs. ozone; PRP vs. placebo	Registered on PROSPERO (CRD42016045410)	Cochrane RoB tool 19	Pain, function, and adverse events	Yes	National Natural Science Foundation of China and Shanghai Youth Science and Technology start-up grants
Simental, 2023	14	PRP vs. placebo	Registered on PROSPERO (CRD42022320169)	Cochrane RoB tool 2.0 21	Pain and function	Yes	No funding
Tan, 2020	26	PRP vs. HA	Not registered	Modification of the tool used by the Cochrane bone, joint, and muscle trauma group 24	Pain, function, adverse events, reintervention rate, C-reactive protein levels, quality of life, satisfaction rate, and return to activities of daily living and sports	Yes	No funding
Tao, 2023	7	Single injection of PRP vs. Multiple injections of PRP	Not registered	Cochrane RoB tool 19	Pain and adverse events	Yes	No funding
Vilchez, 2022	31	PRP vs. HA; PRP vs. corticosteroids; PRP vs. peptide	Registered on PROSPERO (CRD42020202048)	Cochrane RoB tool 2.0 21	Pain and function	Yes	No funding
Xiong, 2023	24	PRP vs. saline solution; PRP vs. HA	Registered on PROSPERO (CRD42022362066)	Cochrane RoB tool 19	Pain, function, and adverse events	Yes	Science and Technology Research Project of Jiangxi Provincial Education
Zhao, 2020	30	PRP vs. saline solution; PRP vs. HA; PRP vs. corticosteroids; PRP vs. acetaminophen; PRP vs. no injection	Registered on PROSPERO (CRD42018100067)	Cochrane RoB tool 19	Pain and function	Yes	National Key Research and Development Program of China; the National Natural Science Foundation of China

Abbreviations: CS: Corticosteroid; HA, hyaluronic acid; NSAIDs, non-steroidal anti-inflammatory drugs; PRISMA, Preferred Reporting Items for Systematic Reviews and Meta-Analyses; PROSPERO, International Prospective Register of Systematic Reviews; PRP: Platelet-rich plasma; RCT, randomized clinical trial; RoB, Risk of Bias tool.

Almost half of the reviews registered their protocol on the International Prospective Register of Systematic Reviews (PROSPERO), although two did not report the registration number. Meta-analyses were conducted in 87% (27/31) of the reviews. The RCT methodological quality tool most used was the Cochrane Risk of Bias Tool (RoB) (68%; 21/31). Regarding financial support, 80.64% (25/31) of the included reviews stated that there were no funding sources nor conflicts of interest.

### Methodological Quality of the Included Systematic Reviews

**Fig. 2:**
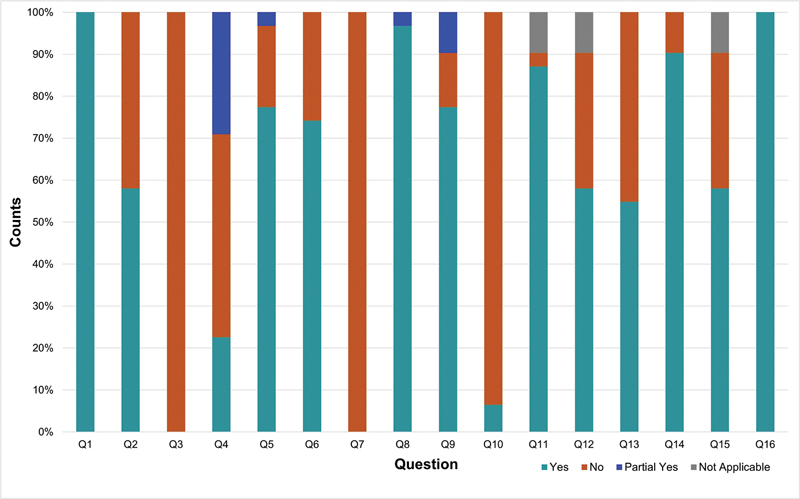
Proportion of categories of methodological quality according to the A Measurement Tool to Assess Systematic Reviews, Version 2 (AMSTAR-2) tool. **Abbreviation:** Q: question. **Notes:** Q1–about the Patient, Intervention, Comparison, Outcome (PICO) strategy; Q2–about protocol a priori; Q3–about study design for inclusion; Q4–about search strategy; Q5–about study selection; Q6–about data extraction; Q7–about list of excluded studies; Q8–about description of the the included studies; Q9–about method to assessing the risk of bias; Q10–about funding of included studies; Q11–about methods for statistical combination; Q12–about impact of risk of bias in individual studies on meta-analysis; Q13–about discussion of risk of bias in individual studies; Q14–about heterogeneity; Q15–about publication bias; and Q16–about conflict of interest.

In the assessment of methodological quality using the AMSTAR-2 tool, 83.8% (26/31) of the systematic reviews were classified as having critically-low quality, followed by 16.13% (5/31) classified as low quality. Details of this assessment are presented in Table 4 , and Fig. 2 shows the frequency of adherence to each AMSTAR-2 item.

**Table 4:** Methodological quality of included systematic reviews (scores on the items of A Measurement Tool to Assess Systematic Reviews [AMSTAR-2] score)

First author, year	Q1	Q2	Q3	Q4	Q5	Q6	Q7	Q8	Q9	Q10	Q11	Q12	Q13	Q14	Q15	Q16	Overall quality
Ali, 2018	Y	N	N	PY	Y	N	N	Y	PY	N	N	N	N	N	N	Y	Critically low
Belk, 2020	Y	N	N	N	Y	Y	N	Y	Y	N	Y	N	N	Y	N	Y	Critically low
Cavazos, 2019	Y	Y	N	Y	Y	Y	N	Y	Y	N	Y	Y	N	Y	N	Y	Critically low
Chen, 2020	Y	N	N	N	Y	Y	N	Y	Y	N	Y	Y	Y	Y	Y	Y	Critically low
Chung, 2018	Y	Y	N	Y	Y	Y	N	Y	Y	N	Y	Y	N	Y	Y	Y	Critically low
Costa, 2022	Y	Y	N	N	Y	Y	N	Y	Y	N	Y	Y	Y	Y	Y	Y	Critically low
Dai, 2017	Y	N	N	Y	Y	Y	N	Y	Y	N	Y	Y	Y	Y	Y	Y	Critically low
Di, 2018	Y	Y	N	N	Y	Y	N	Y	Y	Y	NA	NA	N	N	NA	Y	Critically low
Filardo, 2020	Y	Y	N	Y	Y	Y	N	Y	Y	N	Y	Y	Y	Y	Y	Y	Low
Garcia, 2020	Y	Y	N	N	Y	N	N	Y	N	N	Y	N	N	Y	N	Y	Critically low
Gong, 2021	Y	Y	N	PY	Y	Y	N	Y	Y	N	Y	Y	Y	Y	Y	Y	Low
Hohmann, 2020	Y	N	N	N	Y	Y	N	Y	Y	N	Y	N	N	Y	Y	Y	Critically low
Hong ,2021	Y	N	N	N	N	Y	N	Y	PY	N	Y	N	Y	Y	Y	Y	Critically low
Idres, 2023	Y	N	N	N	Y	Y	N	Y	Y	N	NA	NA	Y	Y	NA	Y	Critically low
Kanchanatawan, 2015	Y	N	N	N	Y	Y	N	Y	N	N	Y	N	Y	Y	Y	Y	Critially low
Kim, 2023	Y	Y	N	PY	Y	N	N	Y	Y	N	Y	Y	N	Y	Y	Y	Critically low
Meheux, 2016	Y	Y	N	N	PY	N	N	Y	N	N	NA	NA	Y	N	NA	Y	Critically low
Nie, 2021	Y	Y	N	N	N	Y	N	Y	Y	N	Y	Y	Y	Y	Y	Y	Critically low
Peng, 2021	Y	Y	N	N	N	N	N	Y	Y	N	Y	Y	N	Y	N	Y	Critically low
Porqueres, 2020	Y	Y	N	N	Y	N	N	Y	Y	N	Y	N	Y	Y	N	Y	Critically low
Sadabad, 2016	Y	N	N	PY	Y	Y	N	Y	Y	N	Y	Y	N	Y	Y	Y	Critically low
Sambe, 2023	Y	N	N	N	N	N	N	Y	Y	N	Y	Y	Y	Y	N	Y	Critically low
Sax, 2022	Y	Y	N	PY	N	N	N	PY	N	N	Y	N	N	Y	N	Y	Critically low
Shang, 2023	Y	N	N	N	Y	Y	N	Y	PY	N	Y	Y	N	Y	N	Y	Critically low
Shen, 2017	Y	Y	N	PY	Y	Y	N	Y	Y	N	Y	Y	Y	Y	Y	Y	Low
Simental, 2023	Y	Y	N	PY	Y	Y	N	Y	Y	N	Y	Y	Y	Y	Y	Y	Low
Tan, 2020	Y	N	N	Y	Y	Y	N	Y	Y	N	Y	Y	Y	Y	Y	Y	Critically low
Tao, 2023	Y	N	N	PY	Y	Y	N	Y	Y	Y	Y	Y	Y	Y	Y	Y	Critically low
Vilchez, 2022	Y	Y	N	Y	Y	Y	N	Y	Y	N	Y	Y	Y	Y	Y	Y	Low
Xiong, 2023	Y	Y	N	Y	Y	Y	N	Y	Y	N	Y	N	N	Y	Y	Y	Critically low
Zhao, 2020	Y	Y	N	PY	N	Y	N	Y	Y	N	Y	N	N	Y	N	Y	Critically low

**Abbreviations:** NA, not applicable; Y, yes; N, no; PY, partially yes; Q: question.

**Notes:** Q1–about the Patient, Intervention, Comparison, Outcome (PICO) strategy; Q2–about protocol a priori; Q3–about study design for inclusion; Q4–about search strategy; Q5–about study selection; Q6–about data extraction; Q7–about list of excluded studies; Q8–about description of the the included studies; Q9–about method to assessing the risk of bias; Q10–about funding of included studies; Q11–about methods for statistical combination; Q12–about impact of risk of bias in individual studies on meta-analysis; Q13–about discussion of risk of bias in individual studies; Q14–about heterogeneity; Q15–about publication bias; and Q16–about conflict of interest.

**Fig. 3:**
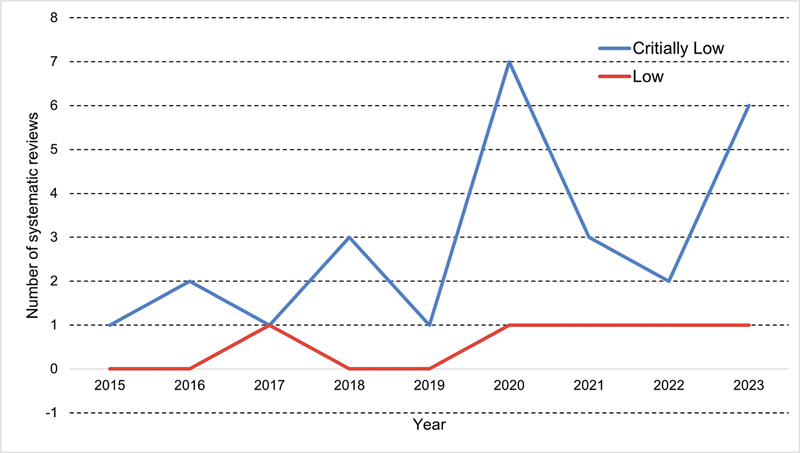
Number of systematic reviews classified as having low and critically-low quality by year.

The current study revealed a significant increase in the number of systematic reviews over the years, with most exhibiting critically-low quality, except for 2017 ( Fig. 3 ).

Among the critical items assessed in the AMSTAR-2, items 4 and 7 emerged as particularly significant in the present evaluation:

- Item 4: 48.3% (15/31) received a “no” response, and 29% (9/31) received a “partially yes” response regarding the performance of a comprehensive and adequate literature search. No systematic review classified as presenting low methodological quality received a negative score in this item, and 57.7% of those classified as “critically low” received “no” (15/31) answers. Reviews that exhibited deficiencies in the literature search were primarily hindered by language restrictions, as evidenced by 38% (12/31) that limited their search only to RCTs written in English. Additionally, 11 reviews that received a poor score on this criterion failed to use trial registry platforms such as ClinicalTrials.gov in their search strategy.
- Item 7: 100% (31/31) did not inform the reasons for excluding RCTs during the study selection process.

### Systematic Reviews that Assessed the Certainty of the Evidence (GRADE approach)

Upon reviewing the AMSTAR-2 tool, we found no mention of assessmets of the certainty of evidence. However, we extracted additional data and found that only 19.35% (6/31) of the included systematic reviews conducted this assessment according to the GRADE approach; however, the complete assessment of each GRADE domain was not fully reported.

## Discussion

Given the significant impact of systematic reviews on clinical recommendations and the development of medical guidelines, they must be conducted using methods that transparently minimize errors and biases. 25 However, despite the widespread dissemination of tools, such as the *Cochrane Handbook of Systematic Reviews* and the PRISMA statement, 14 26 27 28 and the increasing production of systematic reviews over the years, many reviews are still poorly conducted and exhibit a high risk of bias. 29 This finding aligns with the results of the current meta-research study, which observed an increase in reviews on PRP since 2015 but no improvement in their methodological and reporting quality.

As previously mentioned, 83.8% (26/31) of the included systematic reviews received an overall classification of critically-low quality and 16.13% (5/31) were classified as low-quality according to the AMSTAR-2 tool criteria. Considering the critical items, those most inadequately reported are related to search strategies (item 4), with 77,3% of “no” or “partially yes” responses, and the reasons for the exclusion of studies during the selection process (item 7), which was not described in 100% (31/31) of the reviews. Additionally, 42% did not mention the review protocol registry (item 2), 13% did not use the adequate methodological quality tool to assess the risk of bias in RCTs (item 9), 3.5% did not use appropriate statistical methods (item 11), 35.7% did not assess the impact of the RCT's risk of bias on the results (item 12), and 45.2% did not consider the risk of bias in interpreting the data obtained in the discussion section (item 13). Therefore, 32.26% did not meet the item 15 criteria, which involved a plan to investigate publication bias and discuss its impact on the systematic review results. It is important to mention that 100% (31/31) of the included reviews did not report reasons for choosing only RCT to be included in the review (item 3), and 93.5% did not mention the funding sources of their respective included RCTs (item 10). Also, the GRADE approach was used in only 19.3% of the reviews.

Despite methodological recommendations encouraging pre-established protocols for systematic reviews, 29 half of the PRP reviews examined in the present study did not register their protocols beforehand. Without this registration, it is not possible to compare the results obtained with those intended, which compromises the evaluation of possible protocol deviations, 30 and also hinders the to identification of redundancy of systematic reviews on a similar topic without additional contributions. 31

Another topic observed was the absence of a list of excluded references and detailed justifications in all analyzed systematic reviews. This indicates a potential transparency failure in the study selection process. 17 Due to this omission, the interpretation of the results can be underestimated or overestimated. 32

Regarding the design of the studies included in the evaluated systematic reviews (AMSTAR-2, item 3), 100% (31/31) of the reviews did not clarify in the article that this is the best study design to answer research questions. As it is well established, RCTs are considered the gold-standard type of study and the most reliable to test interventions. 33 Therefore, it is assumed that this information is already a consensus, and the authors do not mention it in the text of their reviews.

Almost half of the reviews also conducted search strategies incompletely. In systematic reviews, search strategies involve multiple stages that must be transparent to the reader. Limiting language, mainly selecting only studies written in English, leads to monolingual bias and the potential loss of relevant studies. 32 33 34 35

Even though adherence to the risk of bias tool was high among the systematic reviews on PRP investigated, some studies did not consider the RCTs' risk of bias results in the meta-analysis and discussion, making their conclusions weak. A cross-sectional study 36 showed that efficacy studies with a high risk of bias tend to present higher effect estimates than those with low bias.

No meta-research studies on PRP to treat osteoarthritis were identified. However, a study 37 assessed the methodological quality of systematic reviews in the orthopedic field across the top five impact factor journals between 2006 and 2010. This study 37 revealed that the main areas of deviation were the protocol registry and the assessment of publication bias likelihood, with only 54% of methodological components being fulfilled.

In addition, most reviews (80.6%) did not employ the GRADE approach to assess the certainty of the evidence. Failure to acknowledge evidence certainty and the strength of recommendation through the GRADE approach can lead to misguided guidelines and recommendations, negatively impacting patient health. 38 Due to its critical importance, we suggest that GRADE assessment be included as an item in a future version of the AMSTAR tool.

For the present study, a comprehensive literature search was conducted without date and language restrictions. However, it is possible that eligible reviews were eventually missed. Nevertheless, the decision to include systematic reviews involving only RCTs was due to the excessive number of clinical trials on the topic in question, considering the best primary study to assess the effects of therapeutic interventions. It is important to emphasize that, as meta-research, a sample of studies on the topic was analyzed to generate an overview of the current methodological situation of the published articles.

## Conclusion

The methodological quality of the systematic reviews on the effects of PRP in the treatment of osteoarthritis was classified as having low and critically-low quality through the AMSTAR-2 assessment. Furthermore, most of them did not assess the certainty of the evidence using the GRADE approach. Despite the numerous systematic reviews conducted on this topic over the years, the current study identified that most of these reviews are not rigorously planned and conducted, presenting methodological flaws that could affect the reliability of the clinical findings.
