# Supplementary material for: Methodological Quality of Systematic Reviews on Platelet-Rich Plasma Therapy for Osteoarthritis: A Meta-Research Study
Source: Rev Bras Ortop (Sao Paulo). 2025 Apr 28;60(1):s00451802964. doi: 10.1055/s-0045-1802964 (PMC12037220; doi:10.1055/s-0045-1802964)
Supplement: Related texts

# Research article: Qualidade metodológica das revisões sistemáticas sobre o tratamento de osteoartrite com plasma rico em plaquetas: Um estudo de metapesquisa

**Keywords:** osteoartrite, plasma rico em plaquetas, revisão sistemática

## Abstract

**Objetivo** Avaliar a qualidade metodológica das revisões sistemáticas acerca dos benefícios e malefícios do plasma rico em plaquetas (PRP) no tratamento da osteoartrite.

**Métodos** Realizou-se uma busca abrangente na literatura, e a qualidade metodológica das revisões incluídas foi avaliada usando a ferramenta A Measurement Tool to Assess Systematic Reviews, Version 2 (AMSTAR-2). Além disso, a avaliação da certeza da evidência usando a abordagem Grading of Recommendations Assessment, Development, and Evaluation (GRADE) foi investigada. Ao todo, 31 revisões sistemáticas atenderam aos critérios de inclusão.

**Resultados** A classificação geral de qualidade foi criticamente baixa em quase 84% dos artigos, e baixa em 16,1%. Os critérios metodológicos relatados de forma inadequada com mais frequência estavam relacionados às estratégias de busca, com 77,3% das respostas “não” ou “parcialmente sim”, e os motivos para a exclusão de estudos, que não foram descritos em 100% das revisões. Além disso, 42% não mencionaram o cadastro de seus protocolos, 13% não usaram a ferramenta de qualidade metodológica apropriada para a avaliação do risco de viés dos estudos clínicos incluídos, 45,2% não consideraram o risco de viés na discussão dos resultados, e 32,26% não relataram ou planejaram relatar o viés de publicação. A abordagem GRADE somente foi usada em 19,3% das revisões.

**Conclusão** Apesar do alto número de revisões sistemáticas sobre o tratamento de osteoartrite com PRP, neste estudo de metapesquisa, identificou-se que a maioria não é conduzida adequadamente, e apresenta falhas metodológicas que podem afetar a confiabilidade dos achados clínicos.

## Introdução

As intervenções de saúde frequentemente ganham popularidade e se tornam o foco de ampla investigação científica. Muitas vezes, este é o cenário ideal para a proliferação de sínteses de evidências derivadas dos resultados dos numerosos estudos clínicos publicados sobre a eficácia e a segurança dessas intervenções. Um exemplo notável é a terapia com plasma rico em plaquetas (PRP) 1 , uma injeção que contém alta concentraç ão de plaquetas de um indivíduo, considerada promissora devido ao seu potencial de liberação de fatores de crescimento citocinas e várias substâncias que induzem analgesia, efeitos anti-inflamatórios e anabólicos teciduais no sítio de aplicação, o que pode resultar em uma renovação do tecido danificado. 2 3 4 Além disso, é uma técnica minimamente invasiva e de fácil acesso, que tem recebido atenção significativa nos últimos anos devido ao potencial benefício como tratamento adjuvante para diversas doenças. Em particular, o crescente interesse no uso do PRP no tratamento da osteoartrite é destacado pela importância da inflamação sinovial na fisiopatologia e sua alta prevalência em todo o mundo. 4 5

Dado o aumento significativo de publicações científicas sobre o tema, como esperado, o número de revisões sistemáticas sobre os efeitos do PRP na osteoartrite tem crescido progressivamente nos últimos 10 anos, o que gera uma massa de síntese de evidências que muitas vezes desconsidera os padrões e recomendações metodológicas, o que compromete os resultados. 6 7 Diante desse cenário, os estudos de metapesquisa surgem como uma ferramenta importante para se estabelecer uma conexão entre a boa ciência e sua aplicabilidade na prática clínica. 8 9 Ao reunir e avaliar a adequação metodológica no planejamento, na condução e no relato de diferentes modelos de estudo, a metapesquisa oferece informações sobre o quão comuns e prejudiciais são alguns vieses para um campo de pesquisa. 10 Além de identificar falhas metodológicas produzidas e reproduzidas nas inúmeras revisões sistemáticas sobre PRP no tratamento da osteoartrite, um estudo de metapesquisa pode direcionar esforços futuros para o desenvolvimento de revisões mais pertinentes e de alta qualidade nesta área, o que aumentará a transparência e a reprodutibilidade de seus achados. 11 12

Assim, este estudo de metapesquisa teve como objetivo avaliar a qualidade metodológica de revisões sistemáticas sobre os benefícios e malefícios do PRP em comparação a placebo, outras intervenções, ou nenhuma intervenção no tratamento de pacientes com osteoartrite.

## Materiais e Métodos

Este estudo seguiu a orientação metodológica recomendada para estudos de metapesquisa. 13 Os itens relevantes da declaração Preferred Reporting Items for Systematic Reviews and Meta-Analyses (PRISMA) 14 foram usados para melhorar a qualidade do relato. O protocolo do estudo foi cadastrado na Open Science Framework (disponível em: https://doi.org/10.17605/OSF.IO/NZXT9 ).

## Critérios para a Inclusão de Revisões Sistemáticas

Estudos identificados como revisões sistemáticas de avaliação dos efeitos do PRP no tratamento de qualquer osteoartrite foram elegíveis para inclusão. Apenas aqueles explicitamente rotulados como “revisões sistemáticas” foram considerados, ao passo que protocolos de revisão e revisões sistemáticas que incorporavam delineamentos experimentais observacionais foram excluídos. Também foram excluídas revisões sistemáticas disponíveis apenas como resumos ou em formatos incompletos.

## Fontes de Informação

A identificação das revisões sistemáticas elegíveis foi baseada em uma busca abrangente realizada em 31 de janeiro de 2024 nas seguintes bases de dados eletrônicas: Medical Literature Analysis and Retrieval System Online (MEDLINE, via PubMed), the Cochrane Library (Cochrane Database of Systematic Reviews, CDSR), Excerpta Medica Database (Embase, via Elsevier) e Epistemonikos. Nenhuma restrição quanto à data de publicação ou idioma foi aplicada. As estratégias detalhadas de busca estão apresentadas na Tabela 1 .

**Tabela 1:** Estratégias de busca

Banco de dados	Estratégias de busca	Resultados
Medical Literature Analysis and Retrieval System Online (MEDLINE, via Pubmed)	*#1 “Osteoarthritis” [Mesh] OR Osteoarthritides OR Osteoarthrosis OR Osteoarthroses OR (Arthritis, Degenerative) OR (Arthritides, Degenerative) OR (Degenerative Arthritides) OR (Degenerative Arthritis) OR Arthrosis OR Arthroses* *#2 “Platelet-Rich Plasma” [Mesh] OR (Plasma, Platelet-Rich) OR (Platelet Rich Plasma) OR PRP* *#3 systematic[sb]* *#4 #1 AND #2 AND #3*	221
The Cochrane Library (Cochrane Database of Systematic Reviews, CDSR)	*#1 MeSH descriptor: [Osteoarthritis] explode all trees* *#2 Osteoarthritides OR Osteoarthrosis OR Osteoarthroses OR (Arthritis, Degenerative) OR (Arthritides, Degenerative) OR (Degenerative Arthritides) OR (Degenerative Arthritis) OR Arthrosis OR Arthroses* *#3 MeSH descriptor: [Platelet-Rich Plasma] explode all trees* *#4 (Plasma, Platelet-Rich) OR (Platelet Rich Plasma) OR PRP* *#5 #1 OR #2* *#6 #3 OR #4* *#7 #5 AND #6 In Cochrane Reviews*	7
Excerpta Medica Database (Embase, via Elsevier)	*#1 'osteoarthritis'/exp OR Osteoarthritides OR Osteoarthrosis OR Osteoarthroses OR 'Arthritis, Degenerative' OR 'Arthritides, Degenerative' OR 'Degenerative Arthritides' OR 'Degenerative Arthritis' OR Arthrosis OR Arthroses* *#2 'platelet-rich plasma cell'/exp OR 'Plasma, Platelet-Rich' OR 'Platelet Rich Plasma' OR PRP* #3 ('systematic review' OR 'meta-analysis') AND [review]/lim OR 'meta analysis'/exp OR 'meta analysis' OR 'systematic review'/exp OR 'systematic review' OR 'systematic review (topic)'/exp OR 'systematic review (topic)' OR 'meta analysis (topic)'/exp OR 'meta analysis (topic)' OR 'biomedical technology assessment'/exp OR 'biomedical technology assessment' OR 'network meta-analysis'/exp OR 'network meta-analysis' OR ((systematic* NEAR/3 (review* OR overview*)):ti,ab,kw) OR ((methodologic* NEAR/3 (review* OR overview*)):ti,ab,kw) OR ((quantitative NEAR/3 (review* OR overview* OR synthes*)):ti,ab,kw) OR ((research NEAR/3 (integrati* OR overview*)):ti,ab,kw) OR ((integrative NEAR/3 (review* OR overview*)):ti,ab,kw) OR ((collaborative NEAR/3 (review* OR overview*)):ti,ab,kw) OR ((pool* NEAR/3 analy*):ti,ab,kw) OR 'data synthes*':ti,ab,kw OR 'data extraction*':ti,ab,kw OR 'data abstraction*':ti,ab,kw OR 'handsearch*':ti,ab,kw OR 'hand search*':ti,ab,kw OR 'mantel haenszel':ti,ab,kw OR 'peto':ti,ab,kw OR 'der simonian':ti,ab,kw OR 'dersimonian':ti,ab,kw OR 'fixed effect*':ti,ab,kw OR 'latin square*':ti,ab,kw OR 'met analy*':ti,ab,kw OR 'metanaly*':ti,ab,kw OR 'technology assessment*':ti,ab,kw OR 'hta':ti,ab,kw OR 'htas':ti,ab,kw OR 'technology overview*':ti,ab,kw OR 'technology appraisal*':ti,ab,kw OR 'meta regression*':ti,ab,kw OR 'metaregression*':ti,ab,kw OR 'meta-analy*':ti,ab,kw,ok OR 'metaanaly*':ti,ab,kw,ok OR 'systematic review*':ti,ab,kw,ok OR 'biomedical technology assessment*':ti,ab,kw,ok OR 'bio-medical technology assessment*':ti,ab,kw,ok OR medline:ti,ab,ok OR cochrane:ti,ab,ok OR pubmed:ti,ab,ok OR medlars:ti,ab,ok OR embase:ti,ab,ok OR cinahl:ti,ab,ok OR cochrane OR 'health near/2 technology assessment' OR 'evidence report' OR ((comparative NEAR/3 (efficacy OR effectiveness)):ti,ab,kw,ok) OR 'outcomes research':ti,ab,kw,ok OR 'relative effectiveness':ti,ab,kw,ok OR ((('indirect' OR 'indirect treatment' OR 'mixed-treatment' OR 'bayesian') NEAR/3 comparison*):ti,ab,kw,ok) OR 'meta-analysis'/dm OR 'systematic review'/dm OR ((multi* NEAR/3 treatment NEAR/3 comparison*):ti,ab,kw,ok) OR ((mixed NEAR/3 treatment NEAR/3 ('meta analy*' OR metaanaly*)):ti,ab,kw,ok) OR (umbrella:ti,ab,kw,ok AND review*:ti,ab,kw,ok) OR ((multi* NEAR/2 paramet* NEAR/2 evidence NEAR/2 synthesis):ti,ab,kw,ok) OR ((multiparamet* NEAR/2 evidence NEAR/2 synthesis):ti,ab,kw,ok) OR (('multi paramet*' NEAR/2 evidence NEAR/2 synthesis):ti,ab,kw,ok) *#4 #1 AND #2 AND #3 AND [embase]/lim NOT ([embase]/lim AND [medline]/lim)*	135
Epistemonikos	*((title:(Osteoarthritis OR Osteoarthritides OR Osteoarthrosis OR Osteoarthroses OR (Arthritis, Degenerative) OR (Arthritides, Degenerative) OR (Degenerative Arthritides) OR (Degenerative Arthritis) OR Arthrosis OR Arthroses) OR abstract:(Osteoarthritis OR Osteoarthritides OR Osteoarthrosis OR Osteoarthroses OR (Arthritis, Degenerative) OR (Arthritides, Degenerative) OR (Degenerative Arthritides) OR (Degenerative Arthritis) OR Arthrosis OR Arthroses))) OR abstract:((title:(Osteoarthritis OR Osteoarthritides OR Osteoarthrosis OR Osteoarthroses OR (Arthritis, Degenerative) OR (Arthritides, Degenerative) OR (Degenerative Arthritides) OR (Degenerative Arthritis) OR Arthrosis OR Arthroses) OR abstract:(Osteoarthritis OR Osteoarthritides OR Osteoarthrosis OR Osteoarthroses OR (Arthritis, Degenerative) OR (Arthritides, Degenerative) OR (Degenerative Arthritides) OR (Degenerative Arthritis) OR Arthrosis OR Arthroses)))) AND (title:((Plasma, Platelet-Rich) OR (Platelet Rich Plasma) OR PRP) OR abstract:((Plasma, Platelet-Rich) OR (Platelet Rich Plasma) OR PRP))*	122

## Seleção de Estudos

Duas revisoras (MEOO e MLA) analisaram os títulos e os resumos das referências obtidas a partir da estratégia de busca. Essa triagem inicial foi feita com o auxílio do aplicativo Rayyan (Qatar Computing Research Institute, Doha, Qatar), 15 o que permitiu que o processo de avaliação ocorresse de forma independente. Após essa primeira etapa, o texto completo dos estudos classificados como potencialmente incluídos foi examinado minuciosamente. Quaisquer conflitos em ambas as etapas foram resolvidos por um terceiro revisor. Os estudos excluídos e os motivos para a exclusão estão apresentados na Tabela 2 . Dois revisores independentes usaram um formulário de extração de dados pré-estabelecido para a coleta das informações dos estudos incluídos. Após esse processo, uma terceira revisora (ALCM) resolveu possíveis inconsistências.

**Tabela 2:** Lista de estudos excluídos e motivos para exclusão

**Revisão Sistemática**	**Justificativa**
**Belk, 2023**	Associação de PRP e outra intervenção (CAMO)
**Chou, 2021**	Inclusão de estudos retrospectivos
**Derwich, 2021**	Associação de PRP e outra intervenção
**Evans, 2020**	Inclusão de série de casos
**Hegazy, 2019**	Inclusão de estudos de coorte
**Howlader, 2023**	Inclusão de estudos de coorte
**Kim, 2021**	Inclusão de estudos de coorte
**McLarnon, 2021**	Inclusão de estudos de coorte
**Paget, 2023**	Estudos sem PRP
**Patel, 2022**	Inclusão de estudos prospectivos e retrospectivos
**Tietze, 2015**	Inclusão de série de casos

Abreviaturas: CAMO, concentrado de aspirado de medula óssea; PRP, plasma rico em plaquetas.

## Extração de Dados

Extraímos as seguintes informações das revisões sistemáticas elegíveis: ano de publicação, número de estudos incluídos, aspectos da intervenção e comparadores, protocolo de revisão, ferramenta de avaliação da qualidade metodológica, resultados, a possível condução de meta-análises e as fontes de financiamento. As autoras analisaram várias revisões sistemáticas para avaliar a certeza do corpo da evidência usando a abordagem Grading of Recommendations Assessment, Development, and Evaluation (GRADE) 16 e a adequação do cadastro do protocolo quanto ao viés de relato seletivo.

## Avaliação da Qualidade Metodológica dos Estudos Incluídos

Usamos a ferramenta A Measurement Tool to Assess Systematic Reviews, Version 2 (AMSTAR-2) 17 para a avaliação crítica da qualidade metodológica das revisões sistemáticas. A ferramenta tem 16 itens e, após uma avaliação geral das revisões sistemáticas, cada uma foi categorizada em 4 níveis de qualidade: criticamente baixo, baixo, moderado e alto, dependendo das fraquezas detectadas em itens críticos e não críticos. Duas revisoras independentes (KMMS e MES) conduziram a avaliação com o AMSTAR-2, e uma terceira revisora (EMS) foi consultada em caso de discordâncias.

## Síntese dos Resultados

As características metodológicas das revisões sistemáticas incluídas foram tabuladas e resumidas de forma descritiva numa planilha do programa Microsoft Excel (Microsoft Corp., Redmond, WA, Estados Unidos). Os dados foram expressos por frequências absolutas e relativas.

## Resultados

## Resultados da Busca

Por meio das estratégias de busca, inicialmente foram recuperadas 485 referências. Após eliminar 166 duplicatas, 319 referências passaram por triagem de título e resumo. Assim, 31 revisões sistemáticas atenderam aos critérios de elegibilidade e foram incluídas neste estudo de meta-pesquisa ( Fig. 1 ).

**Fig. 1:** Fluxograma do processo de seleção de estudos. **Abreviatura:** PRP, Plasma rico em plaquetas.

## Características Basais

A Tabela 3 apresenta as características detalhadas das revisões sistemáticas incluídas, 18 19 20 21 22 23 24 que avaliaram entre 3 e 40 ensaios clínicos randomizados (ECRs) sobre os efeitos do PRP em comparação a placebo ou outras terapias. Essas revisões 18 19 20 21 22 23 24 foram publicadas entre 2015 e 2023, com o maior número de publicações em 2020.

**Tabela 3:** Principais características das revisões sistemáticas incluídas

Primeiro autor, ano	ECRs incluídos (n)	Intervenções versus (vs.) comparadores	Protocolo da revisão sistemática	Avaliação da qualidade metodológica	Desfechos	Metanálise	Fontes de financiamento
Ali, 2018	3	PRP vs. AH; PRP vs. PRP + AH	Não cadastrado	Jadad 18	Dor e função	Não	Sem financiamento
Belk, 2020	18	PRP vs. AH	Não cadastrado	Ferramenta RoB, da Cochrane, 19 e Pontuação da Metodologia de Coleman Modificada 20	Dor, função e eventos adversos de LP-PRP vs. LR-PRP	Sim	Sem financiamento
Cavazos, 2019	5	PRP em injeção única vs. PRP em múltiplas injeções	Cadastrado no PROSPERO (CRD42018106429)	Ferramenta RoB, da Cochrane 19	Dor e função	Sim	Sem financiamento
Chen, 2020	14	PRP vs. AH	Não cadastrado	Ferramenta Cochrane RoB 19	Dor, função e eventos adversos	Sim	Sem financiamento
Chung, 2018	5	PRP vs. placebo; PRP vs. AH; PRP vs. soro fisiológico	Número de cadastro no PROSPERO não relatado	Ferramenta RoB, da Cochrane 19	Dor e função	Sim	Sem financiamento
Costa, 2022	40	PRP vs. AH; PRP vs. corticosteroides; PRP vs. soro fisiológico; PRP vs. exercício; PRP vs. medicamentos orais; PRP vs. ozonioterapia; PRP vs. placebo	Cadastrado no PROSPERO (CRD42018093247)	Ferramenta RoB, da Cochrane 19	Dor, função, fracasso terapêutico e eventos adversos	Sim	Sem financiamento
Dai, 2017	10	PRP vs. AH; PRP vs. soro fisiológico; PRP vs. corticosteroides; PRP vs. exercício; PRP vs. sem tratamento	Não cadastrado	Ferramenta RoB, da Cochrane 19	Dor, função e efeitos adversos	Sim	Sem financiamento
Di, 2018	7	PRP vs. AH	Cadastrado no PROSPERO (CRD42016048394)	Ferramenta RoB, da Cochrane 19	Dor e função	Não	Sem financiamento
Filardo, 2020	34	PRP vs. placebo; PRP vs. AH; PRP vs. soro fisiológico; PRP vs. CS; PRP vs. ozônio	Cadastrado no PROSPERO (CRD42019145409)	Ferramenta RoB 2.0, da Cochrane 21	Dor, função e rigidez	Sim	Sem financiamento
Garcia, 2020	7	PRP vs. AH; PRP vs. PRP + AH; PRP vs. soro fisiológico; PRP vs. bupivacaína; PRP vs. sem injeção	Cadastrado no PROSPERO (CRD42020159802)	Pontuação da Metodologia de Coleman 20	Dor, função, e retorno às atividades esportivas e diárias	Sim	Sem financiamento
Gong, 2021	6	PRP vs. AH	Cadastrado no PROSPERO (CRD42020182571)	Ferramenta RoB, da Cochrane 19	Dor, função e eventos adversos	Sim	National Natural Science Foundation of China; Projeto de PesquisaCientífica da Chinese Academy of Traditional Chinese Medicine
Hohmann, 2020	12	PRP vs. AH	Não cadastrado	Ferramenta RoB, da Cochrane 19	Dor e função	Sim	Sem financiamento
Hong, 2021	23	PRP vs. placebo; PRP vs. AH; PRP vs. CS; PRP vs. AINEs orais	Não cadastrado	Versão modificada da Escala de Jadad 22	Dor, função e eventos adversos	Sim	Beijing Municipal Science and Technology Commission; National Natural Science Foundation of China; National Key Research and Development Program of China; Capital Health Research and Development of Special
Idres, 2023	9	PRP vs. corticosteroides	Não cadastrado	Ferramenta RoB, da Cochrane 19	Dor, função e rigidez	Não	Sem financiamento
Kanchanatawan, 2015	9	PRP vs. AH; pRP vs. Placebo	Não cadastrado	PRISMA 14	Dor, função,rigidez e eventos adversos	Sim	Sem financiamento
Kim, 2023	21	PRP vs. AH	Número de cadastro no PROSPERO não relatado	Ferramenta RoB, da Cochrane 19 e Pontuação da Metodologia de Coleman Modificada 20	Dor, função, rigidez e eventos adversos	Sim	Sem financiamento
Meheux, 2016	6	PRP vs. corticosteroides; PRP vs. AH; PRP vs. placebo	Cadastrado no PROSPERO (CRD42014013032)	Pontuação da Metodologia de Coleman Modificada 23	Dor, função e rigidez	Não	Sem financiamento
Nie, 2021	21	PRP vs. corticosteroides; PRP vs. AH; PRP vs. placebo	Cadastrado no PROSPERO (CRD42019122002)	Ferramenta RoB, da Cochrane 19	Dor, eventos adversos e complicações	Sim	Sem financiamento
Peng, 2021	14	PRP vs. AH	Cadastrado no PROSPERO (CRD42022347244)	Ferramenta RoB, da Cochrane 19	Dor, função, e eventos adversos	Sim	Sem financiamento
Porqueres, 2020	4	PRP vs. AH	Cadastrado no PROSPERO (CRD42014010210)	Ferramenta RoB, da Cochrane 19	Dor, função, fatores de crescimento e eventos adversos	Sim	Sem financiamento
Sadabad, 2016	6	PRP vs. AH	Não cadastrado	Ferramenta RoB, da Cochrane 19 e Jadad 18	Dor	Sim	Departmento de Avaliação de Tecnologias de Saúde da Shahid Sadoughi University of Medical Sciences, Yazd
Sambe, 2023	7	PRP vs. AH	Não cadastrado	Ferramenta RoB 2.0, da Cochrane 21	Dor, função e eventos adversos	Sim	Sem financiamento
Sax, 2022	24	PRP vs. Corticosteroides; PRP vs. AH; PRP vs. soro fisiológico e terapia com exercícios	Cadastrado no PROSPERO (CRD42022296909)	Pontuação da Metodologia de Coleman Modificada 23	Dor	Sim	Sem financiamento
Shang, 2023	14	PRP vs. AH	Não cadastrado	Jadad 18	Dor	Sim	Sem financiamento
Shen, 2017	14	PRP vs. AH; PRP vs. ozônio; PRP vs. placebo	Cadastrado no PROSPERO (CRD42016045410)	Ferramenta RoB, da Cochrane 19	Dor, função e eventos adversos	Sim	Bolsas para *start-ups* da National Natural Science Foundation of China e do Shanghai Youth Science and Technology
Simental, 2023	14	PRP vs. placebo	Cadastrado no PROSPERO (CRD42022320169)	Ferramenta RoB 2.0, da Cochrane 21	Dor e função	Sim	Sem financiamento
Tan, 2020	26	PRP vs. AH	Não cadastrado	Modificação da ferramenta usada pelo grupo Cochrane de trauma ósseo, articular e muscular 24	Dor, função, eventos adversos, taxa de reintervenção, níveis de proteína C-reativa, taxa de satisfação, qualidade de vida, e retorno às atividades da vida diária e esportivas	Sim	Sem financiamento
Tao, 2023	7	PRP em injeção única vs. PRP em injeções múltiplas	Não cadastrado	Ferramenta RoB, da Cochrane 19	Dor E eventos adversos	Sim	Sem financiamento
Vilchez, 2022	31	PRP vs. AH; PRP vs. corticosteroides; PRP vs. peptídeo	Cadastrado no PROSPERO (CRD42020202048)	Ferramenta RoB 2.0, da Cochrane 21	Dor e função	Sim	Sem financiamento
Xiong, 2023	24	PRP vs. soro fisiológico; PRP vs. AH	Cadastrado no PROSPERO (CRD42022362066)	Ferramenta RoB, da Cochrane 19	Dor, função e eventos adversos	Sim	Science and Technology Research Project of Jiangxi Provincial Education
Zhao, 2020	30	PRP vs. soro fisiológico; PRP vs. AH; PRP vs. corticosteroides; PRP vs. acetaminofeno; PRP vs. sem injeção	Cadastrado no PROSPERO (CRD42018100067)	Ferramenta RoB, da Cochrane 19	Dor e função	Sim	National Key Research and Development Program of China; National Natural Science Foundation of China

Abreviaturas: CS: Corticosteroide; HA, ácido hialurônico; NSAIDs, anti-inflamatórios não esteroides; PRISMA, Preferred Reporting Items for Systematic Reviews and Meta-Analyses; PROSPERO, International Prospective Register of Systematic Reviews; PRP: Plasma rico em plaquetas; RCT, ensaio clínico randomizado; RoB, ferramenta de risco de viés.

Quase metade das revisões teve seu protocolo cadastrado na plataforma International Prospective Register of Systematic Reviews (PROSPERO), embora duas não tenham informado o número de cadastro. Ao todo, em 87% (27/31) das revisões foram conduzidas meta-análises. A ferramenta de qualidade metodológica do ECR mais utilizada (68%, 21/31) foi a Cochrane Risk of Bias Tool (RoB, The Cochrane Collaboration, Londres, Inglaterra). Em relação ao suporte financeiro, 80,64% (25/31) das revisões declararam não haver fontes de financiamento nem conflitos de interesse.

## Qualidade Metodológica das Revisões Sistemáticas Incluídas

Com a avaliação da qualidade metodológica com a ferramenta AMSTAR-2, 83,8% (26/31) das revisões sistemáticas foram classificadas como de qualidade criticamente baixa, seguidas de 16,13% (5/31) classificadas como de baixa qualidade. Detalhes desta avaliação são apresentados na Tabela 4 , e a Fig. 2 mostra a frequência de adesão a cada item da AMSTAR-2.

**Tabela 4:** Qualidade metodológica das revisões sistemáticas incluídas (pontuação nos itens de A Measurement Tool to Assess Systematic Reviews [AMSTAR-2])

Primeiro autor, ano	Q1	Q2	Q3	Q4	Q5	Q6	Q7	Q8	Q9	Q10	Q11	Q12	Q13	Q14	Q15	Q16	Qualidade geral
Ali, 2018	S	N	N	PS	S	N	N	S	PS	N	N	N	N	N	N	S	Criticamente baixa
Belk, 2020	S	N	N	N	S	S	N	S	S	N	S	N	N	S	N	S	Criticamente baixa
Cavazos, 2019	S	S	N	S	S	S	N	S	S	N	S	S	N	S	N	S	Criticamente baixa
Chen, 2020	S	N	N	N	S	S	N	S	S	N	S	S	S	S	S	S	Criticamente baixa
Chung, 2018	S	S	N	S	S	S	N	S	S	N	S	S	N	S	S	S	Criticamente baixa
Costa, 2022	S	S	N	N	S	S	N	S	S	N	S	S	S	S	S	S	Criticamente baixa
Dai, 2017	S	N	N	S	S	S	N	S	S	N	S	S	S	S	S	S	Criticamente baixa
Di, 2018	S	S	N	N	S	S	N	S	S	S	NA	NA	N	N	NA	S	Criticamente baixa
Filardo, 2020	S	S	N	S	S	S	N	S	S	N	S	S	S	S	S	S	Baixa
Garcia, 2020	S	S	N	N	S	N	N	S	N	N	S	N	N	S	N	S	Criticamente baixa
Gong, 2021	S	S	N	PS	S	S	N	S	S	N	S	S	S	S	S	S	Baixa
Hohmann, 2020	S	N	N	N	S	S	N	S	S	N	S	N	N	S	S	S	Criticamente baixa
Hong, 2021	S	N	N	N	N	S	N	S	PS	N	S	N	S	S	S	S	Criticamente baixa
Idres, 2023	S	N	N	N	S	S	N	S	S	N	NA	NA	S	S	NA	S	Criticamente baixa
Kanchanatawan, 2015	S	N	N	N	S	S	N	S	N	N	S	N	S	S	S	S	Criticamente baixa
Kim, 2023	S	S	N	PS	S	N	N	S	S	N	S	S	N	S	S	S	Criticamente baixa
Meheux, 2016	S	S	N	N	PS	N	N	S	N	N	NA	NA	S	N	NA	S	Criticamente baixa
Nie, 2021	S	S	N	N	N	S	N	S	S	N	S	S	S	S	S	S	Criticamente baixa
Peng, 2021	S	S	N	N	N	N	N	S	S	N	S	S	N	S	N	S	Criticamente baixa
Porqueres, 2020	S	S	N	N	S	N	N	S	S	N	S	N	S	S	N	S	Criticamente baixa
Sadabad, 2016	S	N	N	PS	S	S	N	S	S	N	S	S	N	S	S	S	Criticamente baixa
Sambe, 2023	S	N	N	N	N	N	N	S	S	N	S	S	S	S	N	S	Criticamente baixa
Sax, 2022	S	S	N	PS	N	N	N	PS	N	N	S	N	N	S	N	S	Criticamente baixa
Shang, 2023	S	N	N	N	S	S	N	S	PS	N	S	S	N	S	N	S	Criticamente baixa
Shen, 2017	S	S	N	PS	S	S	N	S	S	N	S	S	S	S	S	S	Baixa
Simental, 2023	S	S	N	PS	S	S	N	S	S	N	S	S	S	S	S	S	Baixa
Tan, 2020	S	N	N	S	S	S	N	S	S	N	S	S	S	S	S	S	Criticamente baixa
Tao, 2023	S	N	N	PS	S	S	N	S	S	S	S	S	S	S	S	S	Criticamente baixa
Vilchez, 2022	S	S	N	S	S	S	N	S	S	N	S	S	S	S	S	S	Baixa
Xiong, 2023	S	S	N	S	S	S	N	S	S	N	S	N	N	S	S	S	Criticamente baixa
Zhao, 2020	S	S	N	PS	N	S	N	S	S	N	S	N	N	S	N	S	Criticamente baixa

**Abreviaturas:** NA, não aplicável; S, sim; N, não; OS, parcialmente sim; Q, questão.

**Notas:** Q1–sobre a estratégia de Paciente, Intervenção, Comparação e Desfecho (Patient, Intervention, Comparison, Outcome, PICO, em inglês); Q2–sobre protocolo *a priori* ; Q3–sobre delineamento experimental do estudo para inclusão; Q4–sobre estratégia de busca; Q5–sobre seleção de estudos; Q6–sobre extração de dados; Q7–sobre lista de estudos excluídos; Q8–sobre descrição dos estudos incluídos; Q9–sobre método para avaliação do risco de viés; Q10–sobre financiamento de estudos incluídos; Q11–sobre métodos para combinação estatística; Q12–sobre impacto de risco de viés em estudos individuais na meta-análise; Q13–sobre discussão de risco de viés em estudos individuais; Q14–sobre heterogeneidade; Q15–sobre viés de publicação; e Q16–sobre conflito de interesses.

**Fig. 2:** Proporção de categorias de qualidade metodológica de acordo com a ferramenta A Measurement Tool to Assess Systematic Reviews, Version 2 (AMSTAR-2). **Abreviatura:** Q: questão. **Notas:** Q1–sobre a estratégia de Paciente, Intervenção, Comparação e Desfecho (Patient, Intervention, Comparison, Outcome, PICO, em inglês); Q2–sobre protocolo *a priori* ; Q3–sobre delineamento experimental do estudo para inclusão; Q4–sobre estratégia de busca; Q5–sobre seleção de estudos; Q6–sobre extração de dados; Q7–sobre lista de estudos excluídos; Q8–sobre descrição dos estudos incluídos; Q9–sobre método para avaliação do risco de viés; Q10–sobre financiamento de estudos incluídos; Q11–sobre métodos para combinação estatística; Q12–sobre impacto de risco de viés em estudos individuais na meta-análise; Q13–sobre discussão de risco de viés em estudos individuais; Q14–sobre heterogeneidade; Q15–sobre viés de publicação; e Q16–sobre conflito de interesses.

Este estudo revelou um aumento significativo no número de revisões sistemáticas ao longo dos anos, com a maioria apresentando qualidade criticamente baixa, exceto em 2017 ( Fig. 3 ).

**Fig. 3:** Número de revisões sistemáticas de qualidade baixa e criticamente baixa por ano.

Entre os itens críticos da AMSTAR-2, os itens 4 e 7 foram particularmente significativos em nossa avaliação:

- Item 4: 48,3% (15/31) tiveram resposta “não”, e 29% (9/31) tiveram resposta “parcialmente sim” em relação à realização de uma busca bibliográfica abrangente e adequada. Nenhuma revisão sistemática classificada como de baixa qualidade metodológica recebeu uma pontuação negativa neste item, e 57,7% (15/31) das classificadas como criticamente baixas receberam “não”. As revisões que exibiram deficiências na busca bibliográfica foram principalmente prejudicadas por restrições de idioma, como evidenciado por 38% (12/31) que limitaram sua busca a ECRs escritos apenas em inglês. Além disso, 11 revisões que receberam uma pontuação baixa neste critério não utilizaram plataformas de cadastro de ensaios, como ClinicalTrials.gov, em sua estratégia de busca.
- Item 7: 100% (31/31) não informaram os motivos da exclusão dos ECRs durante o processo de seleção.

## Revisões Sistemáticas com Avaliação da Certeza da Evidência (Abordagem GRADE)

Ao revisar a ferramenta AMSTAR-2, não encontramos nenhuma menção à avaliação da certeza da evidência. No entanto, extraímos dados adicionais e descobrimos que apenas 19,35% (6/31) das revisões sistemáticas conduziram essa avaliação de acordo com a abordagem GRADE; no entanto, a avaliação completa de cada domínio da GRADE não foi totalmente relatada.

## Discussão

Dado o impacto significativo das revisões sistemáticas nas recomendações clínicas e no desenvolvimento de diretrizes médicas, sua condução deve utilizar métodos que minimizem erros e vieses de forma transparente. 25 No entanto, apesar da grande disseminação de ferramentas, como o *Cochrane Handbook of Systematic Reviews* e a declaração PRISMA, 26 27 28 e a crescente produção de revisões sistemáticas ao longo dos anos, muitas continuam a ser mal conduzidas e têm alto risco de viés. 29 Este achado condiz com os resultados do nosso estudo de metapesquisa, em que se observou um aumento nas revisões sobre PRP desde 2015, mas nenhuma melhora na qualidade metodológica e dos relatos.

Conforme mencionado anteriormente, a classificação geral de qualidade das 31 revisões sistemáticas foi criticamente baixa em 83,8% (26/31) e baixa em 16,13% (5/31) de acordo com os critérios da ferramenta AMSTAR-2. Considerando os itens críticos, aqueles de relatos mais inadequados estão relacionados às estratégias de busca (item 4), com 77,3% de respostas “não” ou “parcialmente sim”, e aos motivos da exclusão de estudos durante o processo de seleção (item 7), que não foram descritos em 100% (31/31) das revisões. Além disso, 42% não mencionaram o cadastro do protocolo da revisão (item 2), 13% não usaram a ferramenta de qualidade metodológica adequada para avaliação do risco de viés dos ECRs (item 9), 3,5% não usaram métodos estatísticos apropriados (item 11), 35,7% não avaliaram o impacto do risco de viés do ECR nos resultados (item 12), e 45,2% não consideraram o risco de viés na interpretação dos dados obtidos na seção de discussão (item 13). Portanto, 32,26% não atenderam aos critérios do item 15, ou seja, um plano para investigação do viés de publicação e discussão de seu impacto nos resultados da revisão sistemática. É importante mencionar que 100% (31/31) das revisões incluídas não relataram os motivos para escolher apenas ECRs para inclusão na revisão (item 3), e 93,5% não mencionaram as fontes de financiamento dos respectivos ECRs incluídos (item 10). Além disso, a abordagem GRADE somente foi usada em 19,3% das revisões.

Apesar das recomendações metodológicas que incentivam protocolos preestabelecidos para revisões sistemáticas, 29 metade das revisões de PRP examinadas neste estudo não cadastraram seus protocolos previamente. Sem esse cadastro, não é possível comparar os resultados obtidos com os pretendidos, o que compromete a avaliação de possíveis desvios de protocolo. 30 A ausência de cadastro também impede a identificação de redundância de revisões sistemáticas sobre um tópico semelhante sem contribuições adicionais. 31

Outro ponto observado foi a ausência de uma lista de referências excluídas e justificativas detalhadas em todas as revisões sistemáticas analisadas. Isso indica uma possível falha de transparência no processo de seleção dos estudos. 17 Esta omissão pode subestimar ou superestimar a interpretação dos resultados. 32

Em relação ao delineamento experimental dos estudos incluídos nas revisões sistemáticas avaliadas (item 3 da AMSTAR-2), 100% (31/31) das revisões não esclareceram que este é o melhor delineamento para responder às questões de pesquisa. Como está bem estabelecido, os ECRs são considerados o padrão-ouro e o mais confiável para testar intervenções. 33 Portanto, presume-se que essas informações já sejam consensuais e os autores não as mencionam no texto de suas revisões.

Quase metade das revisões também usou estratégias de busca de forma incompleta. Em revisões sistemáticas, estratégias de busca envolvem múltiplos estágios que devem ser transparentes para o leitor. Limitar o idioma, principalmente selecionando apenas estudos em inglês, gera um viés monolinguístico com possível perda de estudos relevantes. 32 33 34 35

Embora a adesão à ferramenta de risco de viés tenha sido alta entre as revisões sistemáticas sobre PRP investigadas, alguns estudos não consideraram os resultados de risco de viés dos ECRs na meta-análise nem na discussão, o que torna suas conclusões frágeis. Um estudo transversal 36 mostrou que estudos de eficácia com alto risco de viés tendem a apresentar estimativas de efeito mais altas do que aqueles com baixo viés.

Nenhum estudo de metapesquisa sobre a aplicação de PRP para tratar osteoartrite foi identificado. No entanto, um estudo 37 avaliou a qualidade metodológica de revisões sistemáticas no campo ortopédico nos periódicos com os 5 maiores fatores de impacto entre 2006 e 2010. Este estudo 37 revelou que as principais áreas de desvio foram o cadastro do protocolo e a avaliação da probabilidade de viés de publicação, com cumprimento de apenas 54% dos componentes metodológicos.

Além disso, a maioria das revisões (80,6%) não empregou a abordagem GRADE para avaliação da certeza da evidência. A falha em reconhecer a certeza da evidência e a força da recomendação por meio da abordagem GRADE pode levar a diretrizes e recomendações equivocadas, com impacto negativo sobre a saúde do paciente. 38 Devido à sua importância crítica, sugere-se que a avaliação GRADE seja incluída como um item em uma versão futura da ferramenta AMSTAR.

Neste estudo, foi realizada uma busca abrangente na literatura, sem restrições de data e idioma. No entanto, é possível que algumas revisões elegíveis não tenham sido identificadas. Além disso, devido ao grande número de ensaios clínicos randomizados (ECR) publicados sobre a intervenção em questão, optou-se por analisar exclusivamente revisões sistemáticas que incluíram apenas ECRs, excluindo aquelas que avaliaram estudos observacionais. Essa decisão se baseia no fato de que os ECR representam o delineamento primário mais robusto para avaliar os efeitos das intervenções terapêuticas. É importante ressaltar que, como metapesquisa, uma amostra de estudos sobre o tema foi analisada para se ter uma visão geral da situação metodológica atual dos artigos publicados.

## Conclusão

A qualidade metodológica das revisões sistemáticas sobre os efeitos do PRP no tratamento da osteoartrite foi classificada como baixa e criticamente baixa segundo a ferramenta de avaliação AMSTAR-2. Além disso, a maioria das revisões não avaliou a certeza das evidências pela abordagem GRADE. Apesar das inúmeras revisões sistemáticas conduzidas sobre este tópico ao longo dos anos, este estudo identificou que a maioria delas não é rigorosamente planejada e conduzida, e apresentam falhas metodológicas que podem afetar a confiabilidade dos achados clínicos.
